# The mitochondrial genome of *Atrocalopteryx melli* Ris, 1912 (Zygoptera: Calopterygidae) via Ion Torrent PGM NGS sequencing

**DOI:** 10.1080/23802359.2017.1413307

**Published:** 2018-01-11

**Authors:** Shaolin Xu, Zhaoying Guan, Qi Huang, Lei Xu, Andy Vierstraete, Henri J. Dumont, Qiuqi Lin

**Affiliations:** a Institute of Hydrobiology, Jinan University, Guangzhou, China;; b School of Applied Biology, Shenzhen Institute of Technology, Shenzhen, China;; c South China Sea Fisheries Research Institute, Chinese Academy of Fishery Sciences; Guangdong Provincial Key Laboratory of Fishery Ecology and Environment; Key Laboratory of South China Sea Fishery Resources Development and Utilization, Ministry of Agriculture, Guangzhou, China;; d Department of Biology, University of Gent, Gent, Belgium

**Keywords:** *Atrocalopteryx melli*, damselflies, mitochondrial genome, NGS sequencing, intergenic spacer

## Abstract

The mitochondrial genome of *Atrocalopteryx melli* was sequenced and assembled via Next-Generation Sequencing (NGS) and iteratively assembly process with a reference seed. This genome is 15,562 bp long and A + T biased (71%), with 37 genes arranged in common order of Odonata. All protein-coding genes are initiated by typical “ATN” codon, and 9 genes are terminated with a complete stop codon, except nad4, nad5, cox2, and cox3, which are terminated with an incomplete codon “T(aa)”. The S5 intergenic spacer is absent in this genome, supporting that lacking of S5 as a specific character for damselflies. The A + T rich region of *A. melli* is 267 bp longer than that of *A. atrata*. This mitogenome provides new molecular information for understanding of *A. melli* and *Atrocalopteryx*.

*Atrocalopteryx*, once classified into *Calopteryx*, has already been separated from the genus *Calopteryx* by using molecular phylogenetic method (Dumont et al. 2005). Even in *Atrocalopteryx*, a recent extended phylogeny revealed that one species, *A. oberthueri,* is different from the other species, and it was suggested to be picked out as a new genus (Dumont et al. 2007; Guan et al. 2012). Thus, *Atrocalopteryx* could be split into two genera at least, but its true phylogeny has not been well-resolved yet. More detailed molecular information such as mitochondrial genome and data from more species is required to figure out the reliable phylogeny and infer evolutionary history of *Atrocaloptryx* (Graybeal 1998; Philippe et al. 2011; Schreeg et al. 2016). *Atrocalopteryx melli* (Ris 1912), a large Chinese endemic and beautiful species, is locally abundant in Southern China: Guandong, Fujian, and Guangxi provinces. This species can be found in small shaded headwater streamlets of forests. Here, we sequenced and annotated the mitogenome of *A. melli* to provide molecular information for genetically understanding of the beautiful animal.

A single individual of *A. melli* was sampled from a national natural reserve (a member of IBP, 1979) in Dinghushan mountain, Zhao Qing, Guangdong province (N 23°10′21″ E 112°31′39″), and was preserved in Institute of Hydrobiology, Jinan University, Guangzhou, China. The individual was used for genomic DNA extraction with standard phenol-chloroform method described by Hadrys et al. (1992). Genomic DNA was sequenced on an Ion Torrent PGM sequencer in the Research Group Aging Physiology and Molecular Evolution, Ghent University, Belgium. Skewer v.0.2.1 was used to trim all nucleotide with Phred quality scores under 20 (Jiang et al. 2014). COI sequence of *A. atrata* was used as reference seed for iterative assembly by MITObim v.1.8 (Hahn et al. 2013). Two primers were designed at both ends of the longest assembled sequence, the PCR amplicon was assembled to the original longest sequence using SeqMan v.7.1.0 (Swindell and Plasterer 1997), to get overhang at both ends, which cyclized the final genome with higher reliability. The mitochondrial genome was annotated with the MITOS WebServer (Bernt et al. 2013) and verified via BLAST (Altschul et al. 1990). Transfer RNA genes were then double-checked with tRNAscan-SE v.2 (Lowe and Eddy 1997) and ARWEN v.1.2.3 (Laslett and Canback 2008). Finally, an ultrametric Bayesian phylogenetic tree was reconstructed on 13 PCGs of all available mitogenome of Odonata from NCBI and *A. melli*, following the strategy by Hoi-Sen Yong et al. (2016).

The mitogenome of *A. melli* is 15,562 bp (GenBank accession number: MG011692) in length, with A + T biased base composition: A (41%), T (30%), G (13%), C (16%). Totally, 37 genes are annotated, comprising 13 protein-coding genes (PCGs), 22 tRNA genes, and 2 rRNA genes, ordered in the common arrangement as other Odonata. All PCGs have “ATN” start codons. Nine PCGs are stopped by complete codon: nad3 and cytb with TAG, nad1, nad2, nad4l, nad6, cox1, atp8, and atp6 with TAA. However, nad4, nad5, cox2, and cox3 end with incomplete codon, posttranscriptional polyadenylation could be indispensable for successful translation. tRNA genes have lengths from 64 bp to 72 bp, and they can be folded in the typical cloverleaf structure. The control region (A + T rich: 79%) has a length of 928 bp, shorter than most other Odonata (Lee et al. 2009; Feindt, Herzog, et al. 2016; Feindt, Osigus, et al. 2016; Herzog et al. 2016). Moreover, there are three intergenic spacers with sequence lengths from 9 bp to 16 bp. The S5 spacer is absent in *A. melli*, supporting the earlier finding of a lack of S5 spacer in damselflies (Lin et al. 2010), although this finding could not be universal for that group (Herzog et al. 2016). *Atrocalopteryx melli* and *A. atrata* (NC_027181.1) have the same mitochondrial genes order and composition. However, *A. atrata* has a shorter A + T rich region with length of 661 bp. Our reconstructed phylogenetic tree (Figure 1) showed a sister relationship among *Atrocalopteryx* and *Mnais* +* Vestalis*, which is different from *Vestalis* + (*Atrocalopteryx* +* Mnais*) in the phylogeny of Guan et al. (2012). In contrast to mitochondrial PCGs in the present analysis, nuclear sequences (ITS and 18S rDNA) were used in Guan et al.’s phylogeny, which could be the cause for the above-mentioned difference. Hence, except from getting whole mitochondrial genome and data from more species, combining molecular information from mitochondrion and nuclear might be necessary to fully and reliably resolve phylogenetic relationship within Calopterygidae.

**Figure 1.**
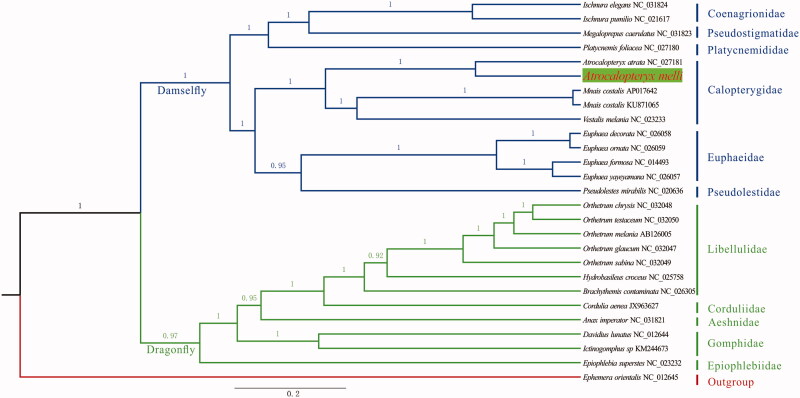
Bayesian phylogenetic tree of all available Odonata mitochondrial genomes together with *Atrocalopteryx melli.* The phylogeny was reconstructed based on 13 mitochondrial PCGs via MrBayes.3.2 with ngen set to 5 million and *Ephemera orientalis* as outgroup.
